# Integrated Microarray-Based Data Analysis of miRNA Expression Profiles: Identification of Novel Biomarkers of Cisplatin-Resistance in Testicular Germ Cell Tumours

**DOI:** 10.3390/ijms24032495

**Published:** 2023-01-27

**Authors:** Jan Roška, João Lobo, Danica Ivovič, Lenka Wachsmannová, Thomas Mueller, Rui Henrique, Carmen Jerónimo, Miroslav Chovanec, Dana Jurkovičová

**Affiliations:** 1 Department of Genetics, Cancer Research Institute, Biomedical Research Center, Slovak Academy of Sciences, Dúbravská cesta 9, 845 05 Bratislava, Slovakia; 2 Cancer Biology and Epigenetics Group, Research Center of IPO Porto (CI-IPOP)/RISE@CI-IPOP (Health Research Network), Portuguese Oncology Institute of Porto (IPO Porto)/Porto Comprehensive Cancer Center (Porto.CCC), Rua Dr. António Bernardino de Almeida, 4200-072 Porto, Portugal; 3 Department of Pathology, Portuguese Oncology Institute of Porto (IPOP), R. Dr. António Bernardino de Almeida, 4200-072 Porto, Portugal; 4 Department of Pathology and Molecular Immunology, School of Medicine and Biomedical Sciences-University of Porto (ICBAS-UP), Rua de Jorge Viterbo Ferreira 228, 4050-313 Porto, Portugal; 5 University Clinic for Internal Medicine IV, Hematology/Oncology, Medical Faculty of Martin Luther University Halle-Wittenberg, 06120 Halle (Saale), Germany

**Keywords:** miRNA microarray, miRNA, cisplatin, testicular germ cell tumour, biomarker

## Abstract

Testicular germ cell tumours (TGCTs) are the most common solid malignancy among young men, and their incidence is still increasing. Despite good curability with cisplatin (CDDP)-based chemotherapy, about 10% of TGCTs are non-responsive and show a chemoresistant phenotype. To further increase TGCT curability, better prediction of risk of relapse and early detection of refractory cases is needed. Therefore, to diagnose this malignancy more precisely, stratify patients more accurately and improve decision-making on treatment modality, new biomarkers are still required. Numerous studies showed association of differential expressions of microRNAs (miRNAs) with cancer. Using microarray analysis followed by RT-qPCR validation, we identified specific miRNA expression patterns that discriminate chemoresistant phenotypes in TGCTs. Comparing CDDP-resistant vs. -sensitive TGCT cell lines, we identified miR-218-5p, miR-31-5p, miR-125b-5p, miR-27b-3p, miR-199a-5p, miR-214-3p, let-7a and miR-517a-3p as significantly up-regulated and miR-374b-5p, miR-378a-3p, miR-20b-5p and miR-30e-3p as significantly down-regulated. In patient tumour samples, we observed the highest median values of relative expression of miR-218-5p, miR-31-5p, miR-375-5p and miR-517a-3p, but also miR-20b-5p and miR-378a-3p, in metastatic tumour samples when compared with primary tumour or control samples. In TGCT patient plasma samples, we detected increased expression of miR-218-5p, miR-31-5p, miR-517a-3p and miR-375-5p when compared to healthy individuals. We propose that miR-218-5p, miR-31-5p, miR-375-5p, miR-517-3p, miR-20b-5p and miR-378a-3p represent a new panel of biomarkers for better prediction of chemoresistance and more aggressive phenotypes potentially underlying metastatic spread in non-seminomatous TGCTs. In addition, we provide predictions of the targets and functional and regulatory networks of selected miRNAs.

## 1. Introduction

Germ cell tumours (GCTs) are heterogeneous neoplasms that originate from germ cells occurring in both male and female patients. Type II testicular GCTs (TGCTs), germ cell neoplasia in situ (GCNIS)-derived, are the most common solid malignancy among young men aged 15–34 years [1,2]. TGCTs account for 90% of all testicular cancers, representing approx. 1.5% of all male neoplasms [3,4] with steadily rising incidence over the last 20 years [5,6,7,8,9,10,11]. The global estimation of the cancer burden in 2018 showed that more than one-third of the 71,000 new cases of testicular cancer occurred in Europe, with the highest predicted rise to appear in East and South European countries [6]. The risk of testicular cancer in white men is about four to five times higher than in black and Asian-American men [12].

Among testicular cancers, three types of GCTs can be classified: type I (teratomas [TE] and yolk-sac tumours [YST]) observed in neonatal boys and children; type II (seminomas and non-seminomas), the most frequent type in young-adults; type III (spermatocytic tumours), which affects mostly men older than 50 years [13]. Within prevalent type II TGCTs, seminoma represents about 60% of the tumours, and non-seminomatous GCTs comprise approx. 40%. Non-seminomatous TGCTs can be further divided according to histology and cellular phenotype into embryonal carcinomas (EC), choriocarcinomas (CC), YST and TE [8,14], with the latest being the most chemoresistant histological subtype [15].

In general, TGCTs respond very well to conventional cisplatin (CDDP)-based treatment, and the overall cure rate of patients even in the metastatic stage is excellent [16,17]. However, about 10% of patients are relapsing or refractory and have poor prognoses. For relapsing patients, high-dose chemotherapy regimens with autologous stem-cell transplants from peripheral blood has appeared promising, but these treatments also increase long-lasting adverse effects on sexual function [18], behaviour [19,20], higher risk of cardiovascular disease [21,22,23], nephrotoxicity [24,25], renal dysfunction [26], neurotoxicity [27] and peripheral neuropathies [28]. In addition, intrinsic or developed drug resistance in relapsing or refractory patients negatively affects treatment outcome and overall costs. Therefore, it is crucial to introduce a precision medicine concept into testicular cancer management. Traditionally, decision-making is based on pre-chemotherapy levels of serum tumour markers, alpha-fetoprotein (AFP), human chorionic gonadotrophin (hCG), lactate dehydrogenase (LDH) and imaging [29,30,31,32]. However, due to low and variable expression, these markers are considered non-specific and unreliable for follow-up and for monitoring advanced stages [32]. Therefore, better and reliable biomarkers are urgently needed to (i) distinguish seminoma from non-seminoma and other non-TGCT testicular malignancies, (ii) stratify clinical stage (CS) I patients indeed benefiting from adjuvant treatment after orchiectomy, (iii) precisely select chemotherapy and monitor treatment response in advanced stages and (iv) predict residual disease after chemotherapy [33].

MicroRNAs (miRNAs) are short RNA molecules able to bind 3’UTRs of numerous mRNA transcripts to regulate their translation [34,35,36]. Several miRNAs have been shown to be deregulated in the pathogeneses of many human cancers [37,38]. Acting as oncogenes or tumour suppressors [39], they regulate tumour development, progression, cell cycle, migration and invasion, and they affect immune or therapy response [40,41]. From a clinical point of view, miRNAs are stable molecules found abundantly in serum [42]; they can also be frequently non-invasively collected from urine or semen and easily detected by PCR. Their potential as biomarkers has already been proven in a variety of malignancies, including in genitourinary cancers [43,44]. In TGCTs, previous studies showed different miRNA expression profiles compared to normal testis and in individual histological subtypes [45]. Ernst and colleagues found the prognostic roles of some miRNAs in seminomatous GCTs by disclosing their ability to identify patients at high risk of relapse and by discriminating metastatic from non-metastatic patients [46]. Deep RNA and miRNA profiling confirmed that RNA and miRNA signals significantly differed between the cases and controls as well as between seminomas and non-seminomas. Circulating RNA and miRNA profiles were shown to be changed during TGCT development according to histology; thus, they may be useful for early detection of a tumour’s type [47]. Identification of the miRNA expression profiles of TGCTs at crucial points of their progression might significantly contribute to precise diagnostics, patient stratification, assessment of staging and subtype classification, but also to drug sensitivity and disease progression prediction, better treatment strategy selection and treatment outcome monitoring [48]. In case of non-seminomas, which present significant intratumoural heterogeneity, miRNA biomarkers can help to characterize not only the primary tumour more precisely, but also the clones responsible for the metastatic phenotype.

In TGCTs, several miRNA clusters have been identified so far to be informative for diagnostics and prognostics, mainly the miR-371-373, miR-302/367 and miR-517/519 clusters [13,45,49,50,51,52]. Expression of miR-371a-3p was extensively analysed as a TGCT biomarker and emerged as the most remarkable non-invasive biomarker for the staging, diagnosis, prognosis and follow-up of TGCT patients [53,54,55]. It is capable of accurately discriminating TGCTs from normal testicular tissue [56,57], of distinguishing individual testicular histotypes and metastatic diseases and of treatment outcome monitoring [58]. However, the specific results of different groups are not uniform, and in some particular cases, they are inconsistent. No significant association between miR-371a-3p expression and clinicopathological parameters, such as stage and prognostic grouping, was found in tumour tissue [55], but higher miR-371a-3p levels were reported in the sera of metastatic patients [56,59]. Furthermore, plasma miR-371a-3p positivity was found only in 13.3% of patients with clinical stage I disease after orchiectomy. No association between plasma miR-371a-3p levels and prognostic factors associated with poor outcomes, such as non-pulmonary visceral metastases or extragonadal GCTs, was revealed [60]. Therefore, miR-371a-3p is considered a biomarker associated with tumour burden [56] rather than with treatment resistance [60]. Overall, cumulative data suggest that miR-371a-3p levels as a biomarker outperform classical serum markers. However, even more sensitive and specific miRNA biomarkers are urgently needed to address all of the particular aspects of TGCTs.

In the present study, we characterized miRNA expression profiles in pairwise combinations of CDDP-sensitive and -resistant TGCT cell lines of different origins and histological types to identify miRNAs potentially serving as predictors of CDDP response in TGCTs. In addition, biomarkers for detection of metastatic potential were also sought based on the use of miRNA expression profiles of metastatic TGCT cell lines. To investigate the clinical relevancies of the selected candidate miRNAs, we validated their expression changes on patient samples. Finally, we performed an in silico analysis of the interactions of these miRNAs to predict their mRNA targets as well as identify their functional and regulatory networks related to CDDP resistance; thus, this will help to diagnose and stratify TGCT patients more precisely and better adjust treatment protocols for both well-responding as well as treatment-resistant patients in crucial time points of the disease’s progression.

## 2. Results

### 2.1. miRNA Expression Profiles in TGCT Cell Lines

TGCT cell lines of different origins and histological types, displaying diverse levels of CDDP sensitivity were used. Cell line selection and categorization was based on the mean 50% and 90% inhibitory CDDP concentrations (IC_50_ and IC_90_ values, respectively) demonstrated in our previous studies [61,62]. As previously reported, the H12.1D, 1411HP and 1777NRpmet (collectively referred to as CDDP-resistant) TGCT cell lines were about 3–21 times more resistant to CDDP than the H12.1, 2102EP and NTERA-2 (collectively referred to as CDDP-sensitive) cell lines [63].

To disclose miRNAs that are differentially expressed in CDDP-resistant TGCT cell lines compared with CDDP-sensitive ones, various pairwise combinations of cell lines were compared. Because the H12.1 and H12.1D TGCT cell lines represent an authentic isogenic pair that shows the highest variance in CDDP sensitivity, priority was given to data acquired for this particular pairwise combination even though the H12.1D cell line was not directly derived to be resistant to CDDP. Other pairs combined non-isogenic cell lines, but the data obtained for them can also be valuable, potentially addressing other aspects of TGCT biology related to CDDP response. In line with this statement, a huge overlap in differentially expressed genes between isogenic and non-isogenic pairwise combinations was obtained [63]. In addition, the metastatic origins and chemoresistant phenotypes of 1411HP and 1777NRpmet revealed miRNAs related to rather intrinsic mechanisms of CDDP resistance. Finally, an analysis comparing all CDDP-resistant TGCT cell lines with all CDDP-sensitive ones may better reflect the histological heterogeneity of the prevalent mixed tumours.

**Figure 1:**
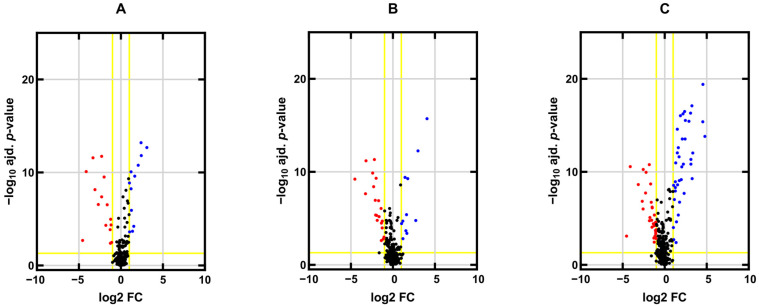
Differentially expressed miRNAs in CDDP-resistant vs. -sensitive TGCT cell lines. Differentially expressed miRNAs in three CDDP-resistant TGCT cell lines (H12.1D, 1411HP and 1777NRpmet) were compared with the CDDP-sensitive H12.1 cell line. Volcano plots represent comparisons of the mean log_2_ FC of miRNA expression between the H12.1D and H12.1 (**A**), 1411HP and H12.1 (**B**) and 1777NRpmet and H12.1 (**C**) TGCT cell lines. Vertical lines correspond to twofold up- and down-regulation. Horizontal lines represent thresholds for the adjusted *p* value < 0.05. Blue and red points in the volcano plot represent twofold up- and down-regulated miRNAs, respectively, that were differentially expressed and statistically significant. Black points represent miRNAs whose expression changes were not statistically significant or did not show >2 FC in expression.

First, we profiled miRNA expression in CDDP-resistant (H12.1D, 1411HP and 1777NRpmet) and CDDP-sensitive (H12.1) TGCT cell lines to identify differentially expressed genes within the H12.1D vs. H12.1, 1411HP vs. H12.1 and 1777NRpmet vs. H12.1 pairwise cell line combinations. Levels of miRNA expression were compared after processing and normalization and correction of the microarray output data. Differences in miRNA expression (represented as the mean fold change, FC) in CDDP-resistant vs. -sensitive TGCT cell lines were considered significant when the *p* value was <0.05. When comparing H12.1D with H12.1, the different expression of 370 miRNAs was found to be statistically significant. Of them, 184 miRNAs were down-regulated and 186 were up-regulated. Notably, 15 miRNAs were down-regulated and 12 miRNAs were up-regulated more than twofold (Figure 1A). When the 1411HP cell line was compared with H12.1, the expression of 428 miRNAs was significantly changed, with 226 and 202 miRNAs being down- and up-regulated, respectively. When a twofold change in expression was set as a criterion, 21 miRNAs were down-regulated, and 14 miRNAs were up-regulated (Figure 1B). The H12.1 vs. 1777NRpmet comparison brought statistical differences in the expression of 457 miRNAs, of which 166 and 291 were up- and down-regulated, respectively, with 38 and 33 miRNAs being up- and down-regulated more than twofold, respectively (Figure 1C).

**Figure 2:**
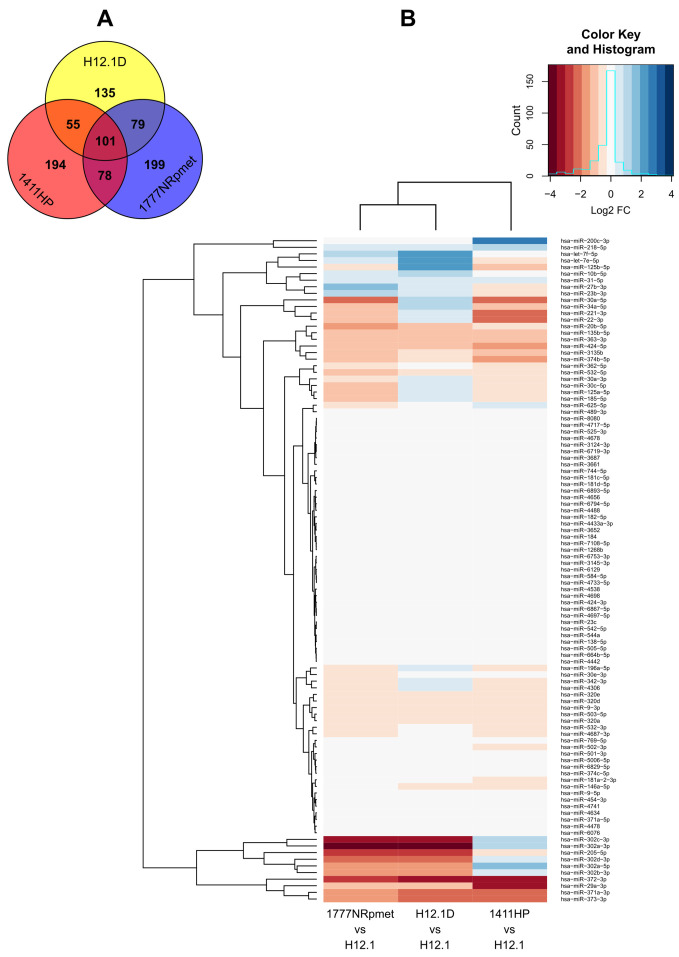
Venn diagram and heatmap of overlapping miRNAs in CDDP-resistant TGCT cell lines. Venn diagram analysis of three CDDP-resistant cell lines (H12.1D, 1411HP and 1777NRpmet) reveals 101 miRNAs whose expression significantly changed (*p* < 0.05) in all three TGCT cell lines when compared to the CDDP-sensitive TGCT cell line H12.1. miRNAs with common changes in expression are presented as overlaps between the three analysed TGCT cell lines. Red colour represents 1411HP, blue represents 1777NRpmet and yellow represents the H12.1D TGCT cell line (**A**). The heatmap illustrates 101 common differentially expressed miRNAs with log_2_ transformed mean FC (*p* < 0.05) in CDDP-resistant TGCT cell lines in comparison with the CDDP-sensitive TGCT cell line H12.1. Red colour indicates decreased relative expression, and blue colour indicates increased relative expression (**B**).

After filtering the data of the miRNA expression changes, we observed intersection or overlap across 101 miRNAs whose expressions significantly changed in all three examined CDDP-resistant cell lines when compared to the H12.1 cell line (Figure 2). We found common expression changes in 79 miRNAs between cell lines H12.1D and 1777NRpmet, 55 miRNAs between H12.1D and 1411HP and 78 miRNAs between metastatic cell lines 1411HP and 1777NRpmet (Figure 2A). This disclosed 135 unique miRNAs in cell line H12.1D, 194 miRNAs in 1411HP and 199 miRNAs in 1777NRpmet, whose expressions significantly changed when compared to the CDDP-sensitive cell line H12.1 (Figure 2A).

When comparing 1411HP to NTERA-2, we found a statistically significant change of expression in 528 miRNAs: 220 up-regulated and 308 down-regulated. Of these, 24 were down-regulated and 17 were up-regulated more than twofold. When comparing 1777NRpmet with NTERA-2, 528 miRNAs showed statistically significant changes in expression. Of these, 333 miRNAs showed down-regulation and 195 showed up-regulation. Twofold changes in statistically significant down-regulation were found in 30 miRNAs, and 47 miRNAs showed twofold up-regulation (Supplementary Materials Figure S1).

We also analysed changes in miRNA expression in two CDDP-resistant isogenic TGCT cell lines with their parental cell lines: H12.1D vs. H12.1 and NTERA-2 vs. NTERA-2 Cis. NTERA-2 Cis showed change in 176 miRNAs, of which 77 were up-regulated and 99 were down-regulated when compared to NTERA-2 (Supplementary Materials Figure S2).

### 2.2. Candidate miRNAs Expression Validation

In order to validate the most relevant candidate miRNAs, we selected 12 miRNAs whose expressions significantly changed in at least two CDDP-resistant TGCT cell lines in comparison to the CDDP-sensitive H12.1 cell line in an expression array. This disclosed four miRNAs which were considered as up-regulated (miR-218-5p, miR-31-5p, miR-125b-5p and miR-27b-3p) in all three CDDP-resistant cell lines and two miRNAs up-regulated in cell lines H12.1D and 1777NRpmet (miR-199a-5p and miR-214-3p) in the original array data set. In addition, we selected four miRNAs which were considered down-regulated (miR-320a, miR-374b-5p, miR-20b-5p and miR-30e-3p) in all three CDDP-resistant cell lines as well as two miRNAs which were down-regulated in cell lines H12.1D and 1411HP (miR-324-5p and miR-378a-3p) in the original array data set. This set of 12 candidate miRNAs was further validated using RT-qPCR in all mentioned TGCT cell lines with the addition of 2102EP and NTERA-2 cell lines that are considered CDDP-sensitive. We also included let-7a-5p, miR-375-5p and miR-517a-3p, which showed the highest up-regulation in cell lines H12.1D, 1411HP and 1777NRpmet in the array data set, respectively.

#### 2.2.1. Up-Regulated miRNAs in TGCT Cell Lines

**Figure 3:**
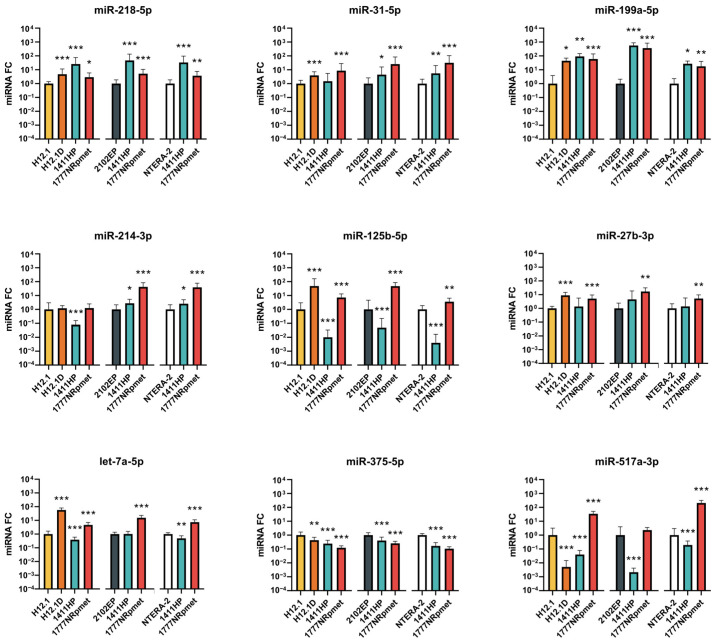
Expression changes of nine candidate miRNAs that were identified as up-regulated in miRNA expression array. CDDP-resistant (H12.1D, 1411HP and 1777NRpmet) and -sensitive (H12.1, 2102EP and NTERA-2) TGCT cell lines were compared. CDDP-resistant cell line H12.1D was compared only with CDDP-sensitive cell line H12.1, as it represents its isogenic pair. Data are presented as mean FC (2^−ΔΔCt^) of CDDP-resistant vs. -sensitive TGCT cell lines from three technical and three biological replicates. Error bars represent upper and lower limits of expression (2^−ΔΔCt ± SDΔCt^). * *p* ≤ 0.05, ** *p* ≤ 0.01, *** *p* ≤ 0.001.

miR-218-5p and miR-199a-5p showed increased expression in all three CDDP-resistant cell lines when compared with all CDDP-sensitive TGCT cell lines (H12.1, 2102EP and NTERA-2). Similarly, with the exception of 1411HP vs. H12.1, increased expression of miR-31-5p was observed. miR-214-3p was up-regulated in the metastatic 1411HP and 1777NRpmet TGCT cell lines only when compared with CDDP-sensitive 2102EP and NTERA-2. In this case, we observed no change in expression in H12.1D or 1777NRpmet and down-regulation in 1411HP when compared with H12.1. A similar trend in expression was observed in the case of miR-125b-5p and miR-27b-3p, where cell lines H12.1D and 1777NRpmet showed increased expression, but 1411HP showed no change of expression or down-regulation (Figure 3). The cell lines H12.1D and 1777NRpmet showed increased expression of let-7a-5p, whereas this miRNA was down-regulated in the 1411HP cell line. All CDDP-resistant cell lines showed decreased expression of miR-375-5p, which did not confirm microarray output, as miR-375-5p was identified with the highest expression in cell line 1411HP. On the other hand, data for miR-517a-3p were confirmed, as expression of this miRNA was increased only in the cell line 1777NRpmet.

#### 2.2.2. Down-Regulated miRNAs in TGCT Cell Lines

**Figure 4:**
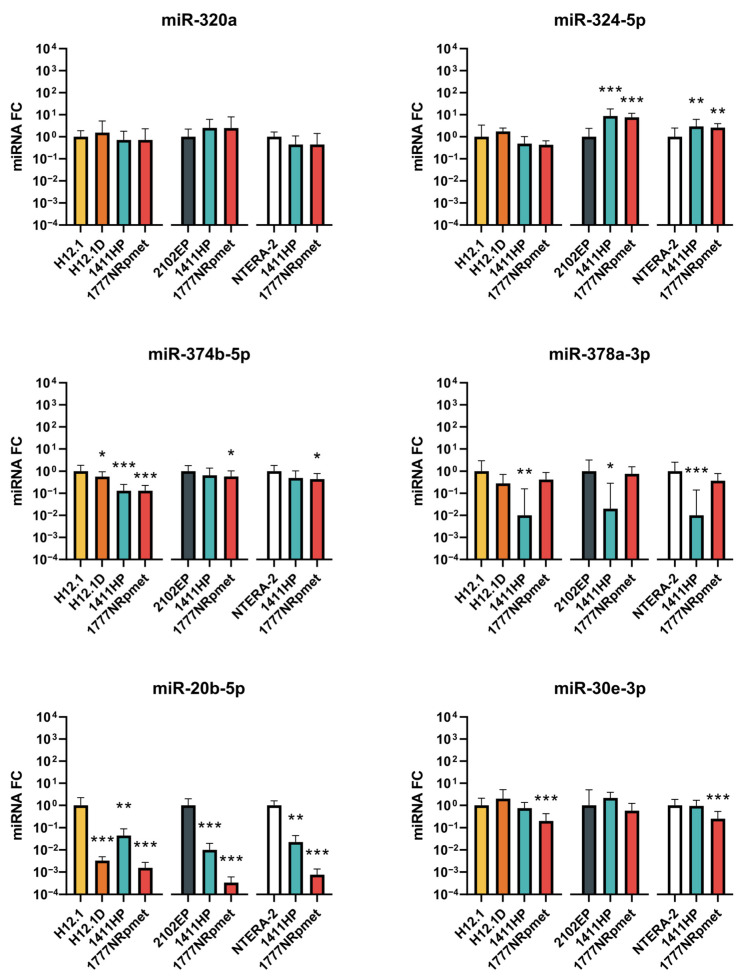
Expression changes of six candidate miRNAs that were identified as down-regulated in miRNA expression array. CDDP-resistant (H12.1D, 1411HP and 1777NRpmet) and -sensitive (H12.1, 2102EP and NTERA-2) TGCT cell lines were compared. CDDP-resistant cell line H12.1D was compared only with CDDP-sensitive cell line H12.1, as it represents its isogenic pair. Data are presented as mean FC (2^−ΔΔCt^) of CDDP-resistant vs. -sensitive TGCT cell lines from three technical and three biological replicates. Error bars represent upper and lower limits of expression (2^−ΔΔCt ± SDΔCt^). * *p* ≤ 0.05, ** *p* ≤ 0.01, *** *p* ≤ 0.001.

We did not observe any significant changes in miR-320a expression in any of the CDDP-resistant cell lines. miR-324 was up-regulated in metastatic CDDP-resistant cell lines 1411HP and 1777NRpmet when compared to the CDDP-sensitive 2102EP and NTERA-2 cell lines. The expression of miR-374b-5p was decreased in all CDDP-resistant cell lines compared to H12.1. However, when compared with 2102EP and NTERA-2, we observed down-regulation only in the 1777NRpmet cell line. Expression of miR-378a-3p was significantly down-regulated only in the CDDP-resistant cell line 1411HP when compared to all CDDP-sensitive cell lines. On the other hand, decreased expression of miR-30e-3p was observed only in the 1777NRpmet cell line. Finally, miR-20b-5p showed significant down-regulation in all CDDP-resistant cell lines when compared with all CDDP-sensitive TGCT cell lines (Figure 4).

#### 2.2.3. Expression of miRNAs in TGCT Patient Samples

**Figure 5:**
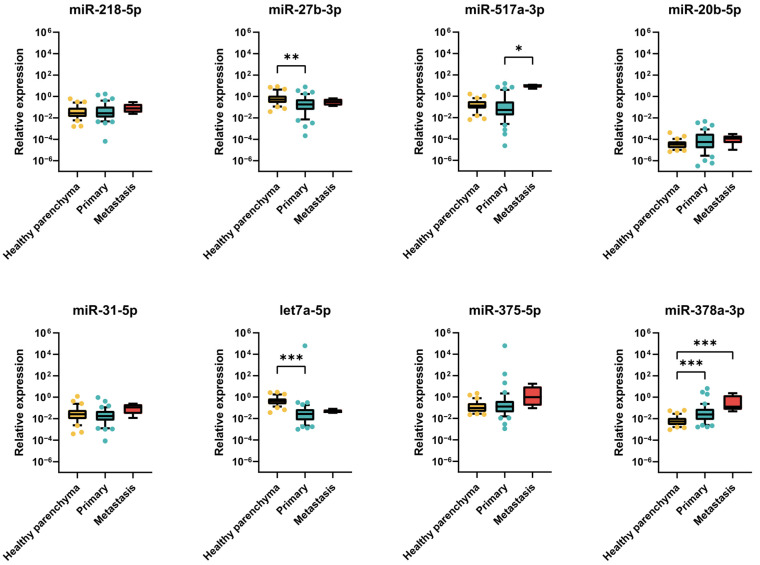
Expression of miRNAs in patient tumour samples. Boxplots show relative miRNA expression (2^−ΔCt^) of miR-218-5p, miR-27b-3p, miR-517a-3p, miR-20b-5p, miR-31-5p, let-7a-5p, miR-375-5p and miR-378a-3p in primary tumour (*n* = 48), metastatic (*n* = 5) and control (normal parenchyma, *n* = 33) samples. Data are presented as medians with interquartile ranges. Whiskers show 10–90 percentile. * *p* ≤ 0.05, ** *p* ≤ 0.01, *** *p* ≤ 0.001.

In order to identify the clinical relevance of the best candidate miRNAs, we further selected miR-218-5p, miR-27b-3p, miR-517a-3p, miR-20b-5p, miR-31-5p, let-7a-5p, miR-375-5p and miR-378a-3p and measured their relative expressions in a set of primary and metastatic TGCT tumour samples in comparison to healthy parenchyma samples. miR-218-5p, miR-31-5p, miR-27b-5p, miR-20b-5p and miR-378a-3p were chosen because their expression changes were confirmed in RT-qPCR validation, whereas miR-517a-3p, let-7a-5p and miR-375-5p were chosen according to their highest individual expressions in CDDP-resistant cell lines in the microarray. We observed the highest median values of relative expression of almost all selected candidate miRNAs, including miR-218-5p, miR-31-5p, miR-20b-5p, miR-378a-3p, miR-375-5p and miR-517a-3p, in metastatic tumour samples when compared with primary and control samples. However, increased expressions of miR-218-5p and miR-31-5p did not prove to be statistically significant (*p* = 0.378 and *p* = 0.249, respectively). We did observe significantly increased relative expression of miR-378a-3p in primary and metastatic tumour samples in comparison to healthy parenchymal tissue (*p* < 0.001 and *p* = 0.0002, respectively) and significantly increased miR-517a-3p expression in metastatic tumour samples in comparison to primary tumour samples, but not against healthy parenchyma (*p* = 0.0449) (Figure 5). We did not observe any statistically significant change in the relative expressions of miR-20b-5p or miR-375-5p.

On contrary, miR-27b-3p and let-7a-3p showed the highest expressions in parenchymal tissue. Elevated expressions of these miRNAs in healthy parenchyma showed statistical significance when compared with primary tumour samples (*p* = 0.0082 and *p* < 0.001, respectively). We did not observe any statistically significant differences in their expressions in metastatic tumour samples (Figure 5).

When individually analysed according to histological classification, we observed increasing expressions of miR-218-5p, miR-31-5p, miR-375-5p and miR-378a-3p as well as miR-20b-5p in advanced, more differentiated subtypes of TGCT tumour samples in comparison to healthy parenchymal tissue. The expressions of miR-517a-3p, miR-27b-3p and let-7a-5p varied among various subtypes. Interestingly, metastatic tumour samples showed elevated expressions of miR-218-5p, miR-517a-3p, miR-375-5p, miR-20b-5p and miR-378a-3p (Supplementary Materials Figure S3). The relative expression levels of miR-218-5p, miR-31-5p, miR-517a-3p and miR-375-5p were measured also in plasma samples of TGCT patients and healthy individuals. The miRNA expression levels, normalized to the geometric mean of miR-191, SNORD44 and SNORD38B in plasma samples, were in line with results obtained in tumour samples and confirmed increased expression in TGCT patients (*n* = 12) compared to healthy donors (*n* = 5) (Supplementary Materials Figure S4).

### 2.3. Predicted Targets and Functional and Regulatory Networks of Candidate miRNAs

In order to predict the candidate mRNA targets of analysed up-regulated miRNAs, we used data from miRNA target prediction databases miRDB (https://mirdb.org/ accessed on 31 August 2022) and TargetScanHuman 8.0. (https://www.targetscan.org/vert_80/ accessed on 31 August 2022). The number of predicted targets for individual miRNAs, along with overlaps or intersections of similar mRNA targets from both databases, is listed in Table 1.

After identifying identical targets, we merged the datasets of the individual predicted targets for miR-218-5p, miR-31-5p and miR-199a-5p, as these miRNAs were up-regulated in all three CDDP-resistant TGCT cell lines after qPCR validation. By overlapping predicted targets of these three miRNAs, we obtained a total number of 1233 targets. Of them, four targets were shared among all three miRNAs (*ZBTB20*, *FZD4*, *CACUL1* and *CEP85L*); miR-218-5p and miR-31-5p shared 26 predicted targets, miR-218-5p and miR-199a-5p shared 36 targets and miR-31-5p and miR-199a-5p shared 15 predicted targets (Supplementary Materials Table S1). As miR-218-5p, miR-31-5p and miR-199a-5p, whose up-regulation was confirmed by RT-qPCR, share a substantial number of predicted targets, it is highly possible that predicted target mRNAs will be the most significantly affected by their up-regulated expression in tumours. The predicted targets *ZBTB20*, *FZD4*, *CACUL1* and *CEP85L* might represent the most promising mRNAs, whose regulation through miRNA is responsible for or contributes to CDDP resistance development. However, these targets are not in direct relationships, and further research is required to determine the impact of these miRNAs on the pathways which concern them. The TGCT cell line H12.1D, besides the mentioned miRNAs, also showed up-regulated expression of miR-125b-5p, miR-27b-3p and let-7a-5p. After including these miRNAs, the total number of 3452 targets was identified, with two of them overlapping throughout five miRNAs (*FZD4* and *CCNJ*), and ten miRNA targets were shared among four miRNAs (*ZBTB20*, *SOX11*, *PRKAA2*, *RNF38*, *RFX3*, *ONECUT2*, *HAPLN1*, *CEP85L*, *EIF5A2* and *ABL2*). For the cell line 1777NRpmet specifically, the predicted targets of miR-517a-3p were also included on top of the abovementioned miRNAs, which resulted in a total number of 3506 targets, and the number of overlapping targets among ≥ 4 miRNAs were similar to the cell line H12.1D.

**Figure 6:**
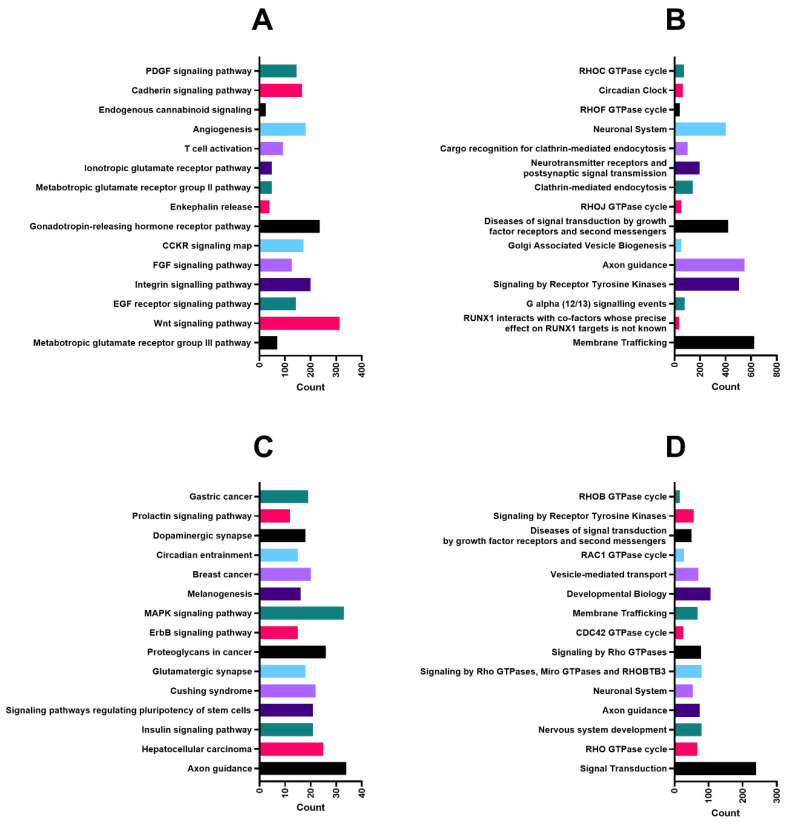
Functional annotation analysis of predicted targets of miR-218-5p, miR-31-5p and miR-199a-5p. PANTHER pathways (**A**) and Reactome pathways (**B**) with functional annotation provided by PANTHER annotation tool; KEGG pathway (**C**) and Reactome pathway (**D**) with functional annotation provided by DAVID annotation tool. Top 15 most significantly (*p* < 0.05) enriched terms are presented as count of genes per annotation.

The predicted targets of miR-218-5p, miR-31-5p and miR-199a-5p were submitted together for functional annotation tools DAVID and PANTHER. Of the total number of 1233 target genes, 1143 were identified in PANTHER, and 1133 were identified in DAVID. The top 15 significantly enriched pathways are presented for two analysis tools from PANTHER (Panther Pathways (Figure 6A) and Reactome (Figure 6B) as well as DAVID or KEGG pathways (Figure 6C) and Reactome (Figure 6D).

Functional annotation analysis of predicted targets for miR-218-5p, miR-31-5p and miR-199a-5p revealed several pathways and cellular processes potentially involved in CDDP-resistant phenotypes, including the Cadherin signalling pathway, angiogenesis, membrane trafficking MAPK and Wnt signalling pathway, RHO GTPase cycle, various pathways associated with cancer and interestingly, multiple times, axon guidance. We suggest that to generate CDDP-resistant phenotypes, examined miRNAs can influence a variety of cellular processes, which are either dependent on stage of differentiation or are influenced by the process of dissemination (in case of 1411HP and 1777NRpmet). Overlaps of the predicted target genes used for functional annotation analysis are listed in Supplementary Materials Table S1.

## 3. Discussion

In our study, multiple comparisons of non-seminomatous CDDP-resistant vs. -sensitive TGCT cell lines were performed to identify novel biomarkers for better prediction of chemoresistance and more aggressive phenotypes in non-seminomatous TGCTs. Despite some slight discrepancies in our results, we propose that miR-218-5p, miR-31-5p, miR-375-5p, miR-517a-3p, miR-20b-5p and miR-378a-3p represent a new panel of biomarkers. In addition, we suggest that the expression levels of miR-27b-3p, let-7a, miR-125b-5p, miR-214-3p and miR-199a-5p can display added predictive value when combined with this panel.

Significant up-regulation of miR-218-5p and miR-31-5p was detected in CDDP-resistant cell lines irrespective of their origin, suggesting their predictive and prognostic value. The expression of miR-218-5p was shown to be down-regulated in most solid tumours while functioning as a tumour suppressor [64], correlating with poor prognoses in non-small cell lung [65], cervical [66] and oral [67] cancers. Conversely, increased expression of this miRNA has been reported in acute lymphocytic leukaemia [68], glioma [69] and metastatic breast [70] and oral [71] cancers. Importantly, no significant increase of miR-218-5p has been reported in chemoresistant TGCTs yet. Our omics data analyses have identified the tumour suppressor Ser/Thr phosphatase PPP2R2A and its regulatory subunit alpha isoform PPP2R5A as direct targets of miR-218-5p. Other up-regulated miRNAs identified herein, miR-31, miR-199a and miR-214, have been reported to target *PPP2R2A, PPP2R5A, PPP2R1A* and *PPP2R1B*, resulting in impaired homologous recombination [72]. Both PPP2R2A and PPP2R5A are activators of the DNA damage response and DNA double-strand break repair pathways after CDDP treatment.

MiR-31-5p was proposed as a biomarker involved in oxaliplatin resistance in colorectal cancer (CRC). Inhibition of miR-31-5p reduced tumour size in a xenograft tumour model, whereas overexpression induced both tumour growth and chemoresistance in cancer cells and tumours. Accordingly, miR-31-5p plays important role in cancer development by directly targeting large tumour suppressor kinase 2 (LATS2) [73]. In head and neck squamous cell carcinoma (HNSCC), both strands of the mature miR-31 were identified as up-regulated in comparison to healthy oral epithelial tissue, suggesting their oncogenic potential. Their inhibition showed attenuation in cell proliferation, invasion and migration, and low expression of miR-31 targets was associated with poor prognosis [74]. MiR-31-5p also induced sorafenib resistance in renal cell carcinoma, resulting in increased colony formation and migration ability both in vivo and in vitro [75]. On the contrary, overexpression of miR-31-5p inhibited cell proliferation, induced apoptosis and increased sensitivity to chemotherapy in triple-negative breast cancer (TNBC) cell lines [76]. It was also down-regulated in CDDP-resistant oesophageal adenocarcinoma (EAC) but up-regulated in 5-fluorouracil-resistant EAC [77].

Expression up-regulation of miR-375-5p reached the highest level in the CDDP-resistant cell line 1411HP in a microarray experiment, although RT-qPCR validation showed significant down-regulation in all CDDP-resistant cell lines instead. Decreased expression of this miRNA is in line with results obtained by Lafin and colleagues [78], who showed poor serum expression of both miR-375-3p and miR-375-5p in TGCT patients with TE, indicating that this miRNA is not a suitable biomarker for this advanced stage of the disease [79], as also confirmed by Lobo and colleagues [79]. Our results showed increased median values of the relative expression of miR-375-5p in patient metastases as well plasma samples when compared to healthy controls, but without any statistical significance.

Our results confirmed that significantly increased expression of miR-517a-3p (together with miR-516b-5p, miR-512-3p, miR-519b, c, d-3p and other members of the cluster) is associated with CDDP resistance. The expression of miR-517a-3p was found most up-regulated in 1777NRpmet when compared to CDDP-sensitive H12.1 and NETRA-2. In clinical content, miR-517a-3p expression significantly discriminated TGCT patients from healthy subjects, and TGCT subjects were metastatic from primary tumour cases. Increased expressions of miR-517a-3p, miR-519a-3p and miR-519c-3p clusters were observed in several tumours and associated with enhanced migration, invasion and poor overall survival (OS) [80,81]. In TGCT Stage I seminoma and mixed tumours with predominant TE component, normal or slightly lower expression of these miRNAs was detected when compared to normal testes. In non-seminomatous and Stage III mixed tumours, these miRNAs displayed high expression. Therefore, miR-517a-3p, miR-519a-3p and miR-519c-3p were suggested to be novel tumour biomarkers for advanced-stage and non-seminomatous TGCTs [82]. Our data shows higher levels of miR517a-3p in the CDDP-resistant 1777NRpmet cell line of metastatic origin, and metastatic tumour patient samples are, at least in part, in line with these observations.

Both miR-378a-3p and miR-20b-5p were identified as down-regulated in CDDP-resistant TGCT cell lines. MiR-378a-3p down-regulation was confirmed in cell line 1411HP. Down-regulation of miR-378a-3p was associated with less favourable OS and disease-free survival in CRC [83,84]. Consistently, down-regulation of miR-378a-3p also correlated with poorer prognoses of ovarian cancer (OC) patients. In OC cell lines, ectopic expression of this miRNA significantly increased the sensitivity of cell lines towards CDDP [85]. The CDDP-sensitizing effect of miR-378a-3p was also confirmed on a glioma model, where down-regulated levels of this miRNA were observed in CDDP-resistant cells [86]. Similarly, in hepatocellular carcinoma, down-regulated miR-378a-3p was associated with higher microvascular density and shorter survival of patients, and its knockdown promoted angiogenesis both in vitro and in vivo [87]. Its tumour-suppressive role might be further confirmed by the fact that it is one of the up-regulated miRNAs in the process of ferroptosis [88]. These findings support our results, which show decreased expression of miR-378a-3p in CDDP-resistant TGCT cell lines. However, we did observe increased expression of miR-378-a-3p in TGCT patients when compared to healthy individuals.

miR-20b-5p was significantly down-regulated in all examined CDDP-resistant TGCT cell lines. A study by Ding and colleagues [89] identified the tumour-suppressive role of miR-20b-5p in lung adenocarcinoma. Its elevated levels inhibited CCND1, a major regulator of cell cycle checkpoints. In CRC, similar effects of miR-20b-5p can be observed. Overexpression of miR-20b-5p inhibited cell cycle, migration and invasion as well as down-regulation of CCND1 [90]. This finding can be further supported by a study by Tang and colleagues [91], who associated elevated expression of miR-20b-5p with suppressed tumourigenicity and attenuated self-renewal capacity of tumour cells. miR-20b-5p mimics decreased the proportion of cancer stem cells and down-regulated the expression of both pluripotency and metabolism proteins. Our results showed increased median values of relative expression of miR-20b-5p in patient metastases when compared to primary tumours and healthy parenchyma, but without any statistical significance.

In our study, miR-27b-3p was significantly up-regulated in the CDDP-resistant cell lines H12.1D and 1777NRpmet, and 1411HP showed non-significant up-regulation when compared to CDDP-sensitive TGCT cell lines. Despite having two mature isoforms, miR-27b-3p was found overexpressed in gastric cancer and noticeably promoted cell proliferation as well as tumour growth by targeting BTG Anti-proliferation factor 2 (BTG2) [92]. MiR-27b-3p was also characterized as an oncogene in BC and CRC by enhancing cyclin D1 expression and thus promoting cell cycle progression and the proliferation of cancer cells [93,94,95,96]. Supporting our result that increased expression of this miRNA is dependent on phenotype and disease progression, miR-27b-3p was also proposed as a metastatic marker, as it was elevated in metastatic group of TNBC compared to the disease-free group [97]. On the contrary, miR-27b-3p was also shown to be a tumour suppressor in gastric [98], lung [99] and breast [100] cancers, further proving its complex role in disease development.

Our results showed the highest expression of let-7a in CDDP-resistant H12.1D when compared to CDDP-sensitive parental H12.1 and significantly increased expression in 1777NRpmet when compared to both CDDP-sensitive lines H12.1 and NTERA-2; however, it did not increase expression in the 1411HP CDDP-resistant cell line nor in patient samples. CDDP-resistant cell line H12.1D is somatic, pre-differentiated, and rather represents a teratomic cell line. Both cell lines H12.1D and 1777NRpmet have lost embryonal markers OCT4, NANOG and LIN28A [101,102]. LIN28A can function as a pluripotency factor and is enriched in stem and progenitor cells and embryonic tissues. This protein can regulate translation via direct binding to mRNAs and by regulating the biogenesis of precursor miRNAs containing a Lin28 recognition motif, principally the let-7 miRNA family [103,104,105]. The CDDP-resistant cell line 1411HP, which is committed to yolk sac differentiation, does express LIN28A like ECs, despite the lack of OCT4 and NANOG embryonic markers, explaining no increase of let-7a in this resistant cell line [101,102]. As overexpression of let-7a resulted in reduced cell growth in the TCam-2 cell line, a tumour suppressor role of this miRNA in seminomas was proposed [106]. According to our results, expression of let-7a was lower in non-seminomatous primary tumours and metastases compared to healthy parenchyma, but still could potentially distinguish CDDP-resistant vs. -sensitive non-seminomatous TGCT cell lines as well as individual resistant non-seminomatous cell lines according to their histology features.

Our results showed up-regulation of miR-125b-5p expression in H12.1D and 1777NRpmet CDDP-resistant cell lines. In another study, miR-125b levels were found to be down-regulated in TGCTs. Overexpression of miR-125b inhibited TGCT growth by targeting CSF1 and CX3CL1, which are known tumour-derived chemokines involved in the recruitment of macrophages to the neoplastic sites [107]. In breast cancer, miR-125b-5p was found to be down-regulated in comparison to normal breast epithelial cell lines, and it was shown that miR-125b-5p suppressed cell proliferation, migration, invasion and colony formation [108]. Similarly, miR-125b-5p acts as a tumour-suppressor in gallbladder cancer, in which its up-regulation promoted cell death in the presence of CDDP and significantly decreased tumour growth. This was probably due to the fact that Bcl2 is a direct target of miR-125b-5p [109]. This was also confirmed in bladder cancer (BC) patient samples, in which lower miR-125b-5p expression significantly correlated with tumour size, distant and lymph node metastasis and shorter progression-free survival. Authors propose that this was due to its effect on regulation of HK2 through suppression of the PI3K/AKT pathway [110]. On the other hand, increased expression of miR-125b-5p in tumour and serum samples was observed in rectal cancer patients who did not respond well to preoperative chemoradiotherapy [111]. The role of miR-125b-5p in carcinogenesis is well-described but complex, and it exhibits tumour-suppressive or oncogenic effects through multiple pathways in tissue-specific manners [112].

Increased expression of miR-214-3p was observed in TGCT cell lines 1411HP and 1777NRpmet. This miRNA is characterized as a major oncomiR, which is up-regulated in various cancer types. In gastric cancer cells, miR-214-3p was shown to induce the Warburg effect by directly targeting genes for the adenosine A2A receptor (*A2AR*) and PR/SET domain 16 (*PRDM16*) and promoting migration and cell proliferation [113]. It was also found to promote transendothelial migration and metastatic dissemination in melanomas and TNBC [114]. Similarly, overexpression of miR-214-3p in NCCIT cells (pluripotent EC cells) reduced the expression of pro-apoptotic protein BCL2-like 11, leading to increased CDDP resistance [115]. On contrary, up-regulation of miR-214 both in papillary thyroid carcinoma and lung cancer cells significantly decreased cell proliferation and resulted in promotion of cell cycle arrest and apoptosis [116,117].

MiR-199a was reported to be down-regulated in TGCTs, partially due to hypermethylation of its promoter. It is co-transcribed with miR-199a-2; both miRNAs are located on chromosome 1 [118,119,120,121,122] and are involved in a complex self-regulatory network along with TP53, DNMT1 and PSMD10, thereby potentially contributing to tumour survival and progression of TGCTs [123]. MiR-199a was down-regulated in malignant tumours and was found to be hypermethylated in malignant testicular tumours. Its expression resulted in suppression of an invasive phenotype [120]. The ectopic expression of miR-199a-5p decreased cell proliferation rate in TNBC [124]. Considering that these two miRNAs are co-expressed and that their function is different among various cancer types, it is possible that their increased expression is a result of the differentiation of TGCTs during malignant progression. This miRNA might further distinguish more advanced phenotypes of tumour cells. The simultaneous down-regulation of miR-199a and miR-214 was observed in the non-seminomatous NTERA-2 cell line when compared to normal human testis cell line Hs 1. Tes. Similarly, in clinical samples, miR-214 expression was significantly down-regulated in EC compared to normal tissues [123]. These results were consistent with the expression levels of both mature forms, miR-199a-3p and miR-199a-5p, in the EC and NTERA-2 cell lines [120]. We propose that the highly complexed regulation of miR-199a and miR-214 expression is tissue-dependent, tumour-dependent, or both, and that the levels of their expression change in chemoresistant vs. chemosensitive TGCT cell lines.

TGCTs are a well-curable malignancy; however, prediction of metastatic and refractory disease, therapy resistance and the risk of relapse remains challenging. The prognosis for TGCT patients depends on the clinical stage at the time of diagnosis; therefore, an early precise diagnosis and treatment can lead to improved outcomes [125]. Current European Association of Urology guidelines still recommend AFP, β-HCG and LDH serum markers for the clinical staging, treatment monitoring and follow-up of TGCT patients [30,126], despite their quite limited informative value [127,128,129,130,131].

To best of our knowledge, our study brings for the first time a panel of differentially expressed miRNAs that discriminate chemoresistant non-seminomatous TGCTs with the potential of metastatic progression from chemosensitive non-seminomatous TGCTs. As evident, our findings are partially in line with previously identified miRNA expression profiles, but they also provide novel miRNAs displaying expression profiles specific for chemoresistant phenotypes and the metastatic potential of non-seminomatous TGCTs. In general, chemoresistant non-seminomatous TGCTs with metastatic parameters show reverted expression profiles of several miRNAs when compared to seminomas or other types of solid malignancies. It is also important to mention that results can differ depending on whether they are compared to healthy controls or parental sensitive tumour cells. This might point to the dual role of miRNAs playing both an oncogenic and tumour-suppressive role in the tumourigenesis of TGCTs in a time-dependent and tissue- or tumour-specific manner. The significant expressional change of miRNA biomarkers might be the important feature to discriminate chemoresistance, worse progression, histological grade or metastatic potential.

Our data, if further validated, may potentially provide tools for the early prediction of TGCT progression and worse prognosis at the time of diagnosis, allowing for better and precise diagnostics and personalized treatment decision-making. It is quite possible that specific miRNAs rule the development and progression of TGCTs and that their significant expression change might reflect crucial points of the disease’s progression. As in a number of similar studies, we are aware of limitations that include the small cohort of patient samples and the retrospective nature of the study. Additional validation studies on larger cohorts of patient samples, including balanced representations of individual TGCT subtypes, primary and metastatic samples and sufficient representations of disease relapse and progression events, are required to fully confirm these findings. However, patients were managed by the same multidisciplinary team of clinicians, assuring homogeneity in classification and treatment decisions. We believe that our results can still be beneficial for summarizing meta-analyses or data processing as well as further studies aimed at the final benefit for the patient.

## 4. Materials and Methods

### 4.1. Cell Cultures

The H12.1, 2102EP, H12.1D, 1411HP and 1777NRpmet TGCT cell lines, provided by our co-author Dr. Thomas Mueller, were grown in RPMI-1640 medium supplemented with 10% foetal bovine serum (FBS), penicillin (100 units/mL) and streptomycin (10 µL/mL). The NTERA-2 and NTERA-2 Cis TGCT cell lines were grown in Dulbecco’s modified Eagle’s medium supplemented with F-10 nutrient mixture (1:1), 10% FBS, penicillin (100 units/mL) and streptomycin (10 µL/mL). Cell lines were cultivated at 37 °C in 5% CO_2_. DNA STR analysis was performed for the individual cell lines used, and the profiles shown in Supplementary Materials Table S2 were found for the H12.1, 2102EP, H12.1D, 1411HP and 1777NRpmet TGCT cell lines using the service provided by the laboratory with certified quality control (GENERI BIOTECH s.r.o., Hradec Králové, Czech Republic). The reference profiles were not found; thus, the match rate could not be determined. However, a comparison of individual profiles showed an 84% identity of an isogenic pair of lines, derived H121.D and parental H12.1, whereas no identity was found in between other cell lines, confirming no cross-contamination of the cell lines used. NTERA-2 and NTERA-2 Cis were obtained after authentication. All cell lines were regularly checked for mycoplasma contamination using MycoSPY^®^ Master Mix—PCR Mycoplasma Test Kit (Biontex Laboratories GmbH, München, Germany).

### 4.2. Patients

Patient tumour samples were retrospectively selected from a cohort of TGCT patients undergoing radical inguinal orchiectomy between 2005 and 2019 at the Portuguese Oncology Institute of Porto (IPO Porto), Portugal. All patients were treated at IPO Porto by the same multidisciplinary team. Additionally, a set of normal testicular parenchyma samples was selected from orchiectomies performed for non-TGCT malignancies (including non-TGCT testicular primary tumours and tumours of the spermatic cord without testicular involvement), all with adequate spermatogenesis. Specimens were routinely fixed in formalin and embedded in paraffin (FFPE) for subsequent staining with Hematoxylin and Eosin (H&E) and histological examination. Sections 8 μm-thick were ordered for RNA extraction. For collection of plasma samples, patients with suspicions of other malignancies were excluded. All blood samples were processed within 4 h maximum from collection. Plasma samples were visually inspected, and no samples had obvious signs of haemolysis. After the collection of patient peripheral blood into EDTA-containing tubes, plasma was separated by centrifuging at 2500 rpm for 30 min at 4 °C and subsequently stored at −80 °C in the IPO Porto’s Biobank [132]. The same protocol was adopted to obtain plasma samples from healthy control donors at the Biomedical Research Center of the Slovak Academy of Sciences, Bratislava, Slovakia. All blood samples coincided with routine determinations of classical serum markers. The investigation of all used patient samples has been approved by the Ethics Committee (CES-IPO-12-018) of the Portuguese Oncology Institute of Porto, Portugal. All procedures were in accordance with the ethical standards of the institutional or national research committee (or both) and with the 1964 Helsinki declaration and its later amendments or with comparable ethical standards. All histological material as well as the clinical and histopathological data of the patients were reviewed by the same TGCT-dedicated pathologist according to the most recent 2016 World Health Organization classification (the cohort’s full characteristics are reported in [133]). Clinical charts were also reviewed, and patients were staged according to the most recent American Joint Committee on Cancer (AJCC) 8th edition staging manual [133]. The characteristics of the TGCT patients used in this study are provided in Supplementary Tables S3 and S4.

### 4.3. RNA Extraction

To extract the total RNA from cell cultures, TRI Reagent solution (Life Technologies) was used. The total RNA was quantified using a MaestroNano Spectrophotometer (Applied Biological Materials Inc., Richmond, Canada) and a Qubit fluorometer (Qubit^®^ RNA HS Assay Kit, Life Technologies, Frederick, MD, USA). RNA integrity was assessed using the Agilent 2100 bioanalyzer (Agilent, Palo Alto, CA, USA). Extracted RNA was used for microarray analysis and RT-qPCR validation.

To extract the total RNA from patient tumour samples, a FFPE RNA/DNA Purification Plus Kit (Cat. 54300, Norgen, Thorold, ON, Canada) was used according to the manufacturer’s protocol. RNA quantification and purity were assessed in a NanoDrop^TM^ Lite Spectophotometer (Cat. ND-LITE, Thermo Scientific^TM^, Waltham, MA, USA). The total RNA from patient plasma samples was extracted from 300 μL of plasma using TRI Reagent^®^BD for processing whole blood, plasma, or serum (Life technologies, Frederick, MD, USA). Total RNA was quantified using the NanoDrop ND-1000 Spectrophotometer (Thermo Scientific, Waltham, MA, USA) or MaestroNano Spectrophotometer (Applied Biological Materials Inc., Richmond, Canada). Extracted RNA was further used for RT-qPCR validation.

### 4.4. miRNA Microarray Profiling

miRNA expression profiling was performed using 100 ng of the total RNA, which was processed using the Agilent miRNA Complete Labelling and Hyb Kit (5190-0456, Santa Clara, CA, USA) according to the manufacturer ’s protocol (Version 4.1, October 2021). Briefly, after dephosphorylation of the 3 ’ ends of RNA molecules, the Cyanine3-pCp was ligated to the 3 ’ end. Next, the samples were dried, suspended in the hybridization mix and loaded onto the SurePrint Human miRNA Microarray, Human miRNA Microarray, Release 21.0 (070156), 8 × 60 K slide (G4872A-070156). The loaded slide, placed in the Agilent Microarray Hybridization Chamber (G2534), was incubated for 20 h at 55 °C/20 rpm in the Agilent hybridization oven (G2545A). After washing with the Gene Expression Wash Buffers 1 and 2 (5188-5327), the washed and dried slide was scanned in the Agilent SureScan Dx Microarray Scanner (Santa Clara, CA, USA) with a resolution of 3 µm. The resulting images of individual arrays were extracted with the Mapix 9.1.0 software (Innopsys Inc., Carbonne, France) and exported as GPR files. GPR files were imported into the R workspace via the read. The Maimages () function in the limma package was used and transformed into the “uRNAList” format to enable the use of functions from the R package “AgiMicroRna”. Subsequently, without background subtraction, data were quantile-normalized between arrays by using the function rmaMicroRna () in the AgiMicroRna package. Finally, standard limma procedure was applied to the normalized data to analyse differential expression between studied groups.

To determine miRNA expression from GPR files, correction of the background- and signal-to-noise ratio was performed to output values and probe signal summarization to the level of individual gene expression was applied. In the next step, the corrected differentially expressed miRNAs were identified by using the limma package, and FCs of miRNA expression in CDDP-resistant vs. -sensitive TGCT cell lines were calculated, along with average expression and *p* values, in order to identify difference in miRNA expression in CDDP-resistant TGCT cell lines.

For result interpretation, we considered miRNA expression to be up-regulated when the ratio in the mean miRNA expression or FC of a CDDP-resistant vs. -sensitive TGCT cell line was >1, and down-regulated when the mean FC between a CDDP-resistant and -sensitive TGCT cell line was <1. This is due to the fact that after microarray visualization and correction to background noise, we compared the corrected resulting signals (representing miRNA expression in samples) as the proportion or difference in average expression in the CDDP-resistant TGCT cell line vs. the CDDP-sensitive one (H12.1).

### 4.5. RT-qPCR Validation

The expression of selected up- and down-regulated miRNAs was evaluated using RT-qPCR. The expression of mature miRNAs in TGCT cell lines, patient tumour and plasma samples was determined using a RevertAid First Strand cDNA Synthesis Kit (Thermo Scientific, Waltham, MA, USA) supplemented with poly(A)polymerase (Takara, Japan) and ATP (Sigma, Germany). Briefly, for cDNA synthesis, 100 ng of the total RNA in a final volume of 20 μL, including 4 μL of 5 × MuLV reaction buffer, 0.1 mM of ATP, 1 μM of RT-primer, 0.1 mM of each deoxynucleotide (dATP, dCTP, dGTP and dTTP), 100 units of MuLV (Murine Leukaemia Virus) reverse transcriptase and 1 unit of poly(A)polymerase, was incubated at 42 °C for 1 h, followed by enzyme inactivation at 95 °C for 5 min. The sequence of the oligo-d(T)/adapter primer was 5′-CAGGTCCAGTTTTTTTTTTTTTTTVN, where V is A, C and G and N is A, C, G and T (IDT, Leuven, Belgium). In each plasma sample, the cDNA yield was quantified using either the NanoDrop ND-1000 Spectrophotometer (Thermo Scientific, Waltham, MA, USA) or MaestroNano Spectrophotometer (Applied Biological Materials Inc., Richmond, Canada) to use equal amounts of cDNA (300 ng in 1 μL) for RT-qPCR. RT-qPCR detection and quantification of mature forms of selected miRNAs was performed using SYBR Premix Ex Taq II (Tli RNaseH Plus), ROX plus (Takara, Japan) and miRNA-specific primers (Table 2). qPCR was performed using ARIA (Agilent) and BIOER, LineGene 9660 RT-qPCR System (Hangzhou Bioer Technology Co., Ltd., Hangzhou, China) at the following settings: 95 °C for 5 min, followed by 40 cycles at 95 °C for 20 s and 60 °C for 50 s, followed by a melt cycle. Each amplification of mature miRNA was done in triplicate. Ct values were normalized against reference controls SNORD38B, SNORD44, SNORD66 and miR-191 and miR-93, respectively. After testing the expressions of several reference control genes, these were selected due to the most stable expressions in TGCT cell lines, patient samples and healthy controls. The geometric means of the Ct values of a minimum of three housekeepers was used for expression data normalization.

### 4.6. miRNA Target Prediction and Functional Annotation Analysis of Predicted Targets

To examine potential impact of up-regulated miRNAs in CDDP-resistant cell lines on gene expression and translation, we used the miRDB—MicroRNA Target Prediction Database [134,135] and TargetScanHuman 8.0 [136,137,138,139,140,141] databases to identify candidate genes that might be regulated by differentially expressed miRNAs. The list of obtained genes from both databases was then processed, and overlaps or intersections of the same predicted targets or genes were later subject to functional annotation analysis.

The list of the predicted targets of the miRNAs, which were up-regulated in all CDDP-resistant TGCT cell lines (miR-218-5p, miR-31-5p, and miR-199a-5p), as well as miRNAs which were up-regulated individually or specifically in CDDP-resistant cell lines (miR-125b-5p, miR-27b-3p and let-7a-5p for H12.1D with the addition of miR-517a-3p for cell line 1777NRpmet) were both separately as well as collectively submitted for functional annotation analysis using the DAVID Bioinformatics Resources tool [142,143] and PANTHER Classification System [144,145,146,147]. The putative pathways were annotated using the Kyoto Encyclopedia of Genes and Genomes and Reactome in DAVID, and Reactome and Panther pathways in PANTHER. A pathway was considered to be significantly enriched if it passed a threshold of three genes per annotation term and presented a *p* value <0.05 (Benjamini for DAVID and Fisher’s Exact test for PANTHER annotation tools) with false discovery rate (FDR) correction. All analyses in which the mentioned databases were used were performed in the time period from May to August 2022.

### 4.7. Statistical Analysis

For statistical analysis of the miRNA expression data, SigmaPlot 12.5 and Prism GraphPad 8.4.3 were used. Normality of distribution was tested by using the Shapiro-Wilk test. Relative quantification of the miRNA expression was calculated with the 2^−ΔΔCt^ method, which represents relative FCs of expression. Therefore, ΔΔCt = ΔCt (CDDP-resistant cell line) − ΔCt (CDDP-sensitive cell line). Analysis of the significance of FC in the miRNA expression between the studied groups was applied to the ΔCt values. If normally distributed, miRNA expression data were tested by using an analysis of variance (ANOVA) with Bonferroni’s post-hoc test for multiple comparisons. If data were normally distributed but did not pass Levene’ test for equality of variances, a one-way ANOVA was used with Tamhane’s T2 test for multiple comparisons. If the data were non-normally distributed, a Kruskal-Wallis one-way ANOVA with Dunn’s test for multiple comparisons was used. All tests were two-tailed. For all analyses, *p* < 0.05 was considered statistically significant (* *p* ˂ 0.05; ** *p* ˂ 0.01, *** *p* ˂ 0.005). Graphic output in the form of a heatmap for miRNA microarray analysis was performed using RStudio (RStudio, Inc., Boston, MA, USA, version 1.2.1335). The cut-off of significantly up- or down-regulated miRNA was selected according to their log FCs and adjusted *p* values in the above-mentioned software and imported into RStudio.

## Figures and Tables

**Table 1:** List of predicted target genes of differentially expressed miRNAs.

miRNA	TargetScanHuman Predicted Targets [*n* (Conserved Sites; Poorly Conserved Sites)]	miRDB Predicted Targets (*n*)	Overlap of the Same Target Genes (*n*)
miR-218-5p	1102 (1262; 453)	1084	686
miR-31-5p	477 (510; 228)	595	221
miR-199a-5p	634 (723; 287)	562	326
miR-125b-5p	931 (1038; 452)	925	569
miR-27b-3p	1421 (1613; 762)	1497	895
let-7a-5p	1207 (1386; 243)	990	755
miR-517a-3p	557	58	54

**Table 2:** List of primers used for qPCR validation of miRNAs’ expressions.

miRNAs	Forward (5′-′3)	Reverse (5′-′3)
miR-218-5p	CGCAGTTGTGCTTGATCTAAC	CAGGTCCAGTTTTTTTTTTTTTTTACAT
miR-31-5p	GCAGAGGCAAGATGCTG	TCCAGTTTTTTTTTTTTTTTAGCTAT
miR-199a-5p	GCAGCCCAGTGTTCAGA	CCAGTTTTTTTTTTTTTTTGAACA
miR-214-3p	GCAGACAGCAGGCACA	AGTTTTTTTTTTTTTTTACTGCCT
miR-125b-5p	GCAGTCCCTGAGACCCTAA	TCCAGTTTTTTTTTTTTTTTTCACA
miR-27b-3p	GCAGTTCACAGTGGCTAAG	CCAGTTTTTTTTTTTTTTTGCAG
miR-320a-5p	GCGCAGGCCTTCTCTT	TCCAGTTTTTTTTTTTTTTTGGAA
miR-324-5p	GCAGCGCATCCCCTA	CAGTTTTTTTTTTTTTTTCACCAAT
miR-374b-5p	CAGCGCAGATATAATACAACCT	GGTCCAGTTTTTTTTTTTTTTTCACTT
miR-378a-3p	GCAGACTGGACTTGGAGT	CCAGTTTTTTTTTTTTTTTGCCTT
miR-20b-5p	CGCAGCAAAGTGCTCATA	CCAGTTTTTTTTTTTTTTTCTACCT
miR-30e-3p	CGCAGCTTTCAGTCGGA	TCCAGTTTTTTTTTTTTTTTGCTGT
let-7a-5p	CGCAGTGAGGTAGTAGGT	GTCCAGTTTTTTTTTTTTTTTAACTATA
miR-375-5p	GGCGACGAGCCCCTC	CAGTTTTTTTTTTTTTTTGGTTTGT
miR-517a-3p	GCAGATCGTGCATCCCTTTA	GGTCCAGTTTTTTTTTTTTTTTACAC
**Housekeepers**		
SNORD44	CAGCCTGGATGATGATAAGCAAAT	GGTCCAGTTTTTTTTTTTTTTTAGTCAGTT
SNORD38B	GCAGCTCAGTGATGAAAACTTTGT	AGGTCCAGTTTTTTTTTTTTTTTTCTCCT
SNORD66	GCAGTTCCTCTGATGACTTCCT	GTCCAGTTTTTTTTTTTTTTTTTCCTCAGA
miR-191-5p	GCAGCAACGGAATCCCAA	CCAGTTTTTTTTTTTTTTTCAGCT
miR-93-5p	CGCAGCAAAGTGCTGTT	TCCAGTTTTTTTTTTTTTTTCTACCT

## Data Availability

Rough data available on request.

## Supplementary Materials

The following supporting information can be downloaded at: https://www.mdpi.com/article/10.3390/ijms24032495/s1.
