# Supplementary material for: Integrated Microarray-Based Data Analysis of miRNA Expression Profiles: Identification of Novel Biomarkers of Cisplatin-Resistance in Testicular Germ Cell Tumours
Source: Int J Mol Sci. 2023 Jan 27;24(3):2495. doi: 10.3390/ijms24032495 (PMC9916636; doi:10.3390/ijms24032495)
Supplement: Supplementary file 1 [file ijms-24-02495-s001.zip › ijms-2127050-supplementary.pdf]

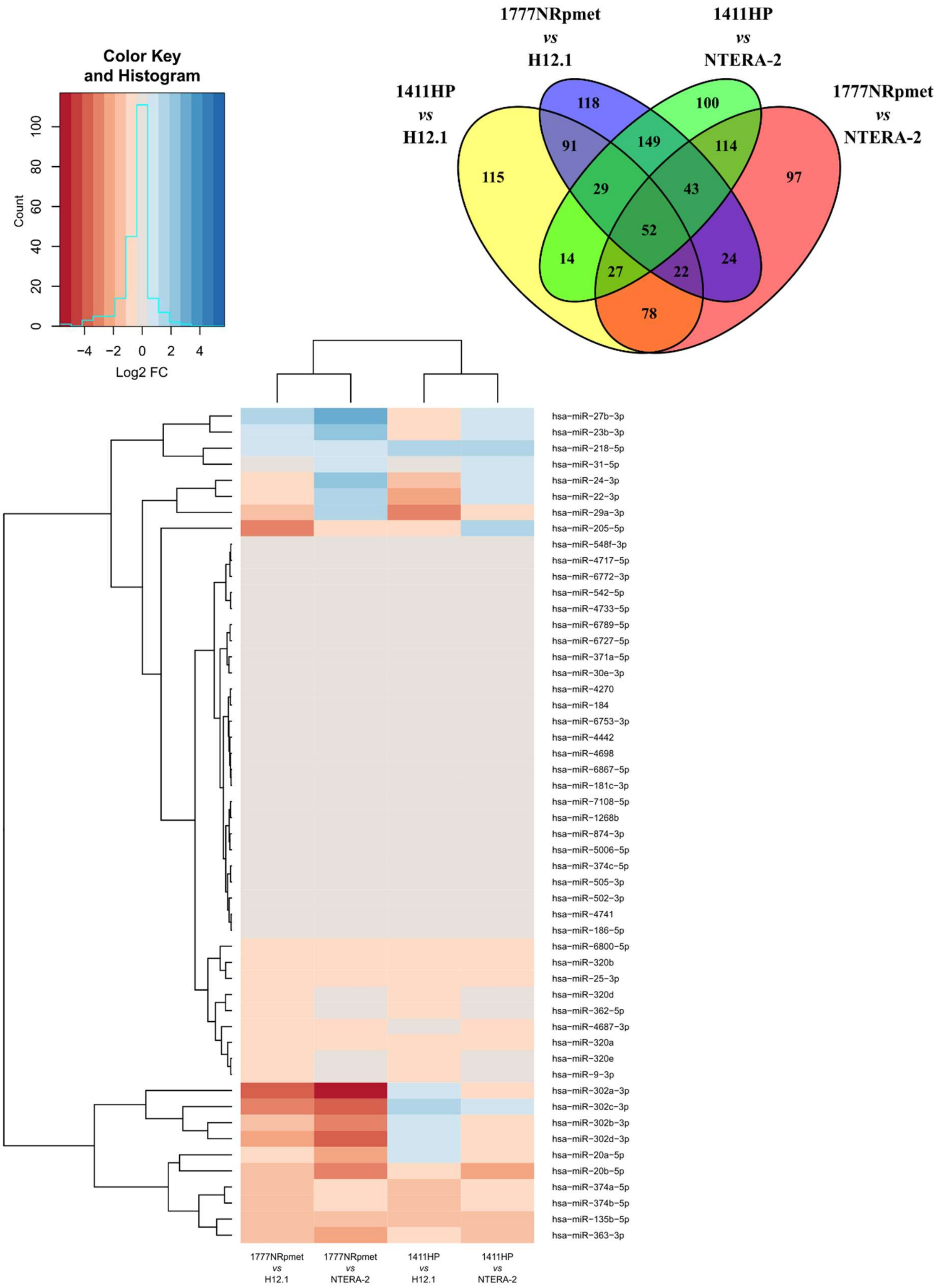

Supplementary Figure S1: Venn diagram and heatmap of overlapping miRNA in CDDP-resistant vs. -sensitive TGCT cell lines. Venn diagram analysis of two CDDP-resistant cell lines reveals 52 miRNAs whose expression was significantly changed ($p < 0.05$) in 1411HP and 1777NRpmet when pairwise combined with H12.1 and NTERA-2 cell lines. The common miRNAs are presented by overlapping overexpressed miRNAs in analysed TGCT cell lines. 1411HP vs. H12.1 (yellow), 1777NRpmet vs. H12.1 (blue), 1411HP vs. NTERA-2 (green) and 1777NRpmet vs. NTERA-2 (red). Heatmap illustrates 52 common differentially expressed miRNA with log2 transformed mean FC ($p < 0.05$) in CDDP-resistant TGCT cell lines 1411HP and 1

1777NRpmet in comparison with -sensitive TGCT cell lines H12.1 and NTERA-2. Red colour indicates decreased relative expression and blue colour indicates increased relative expression.

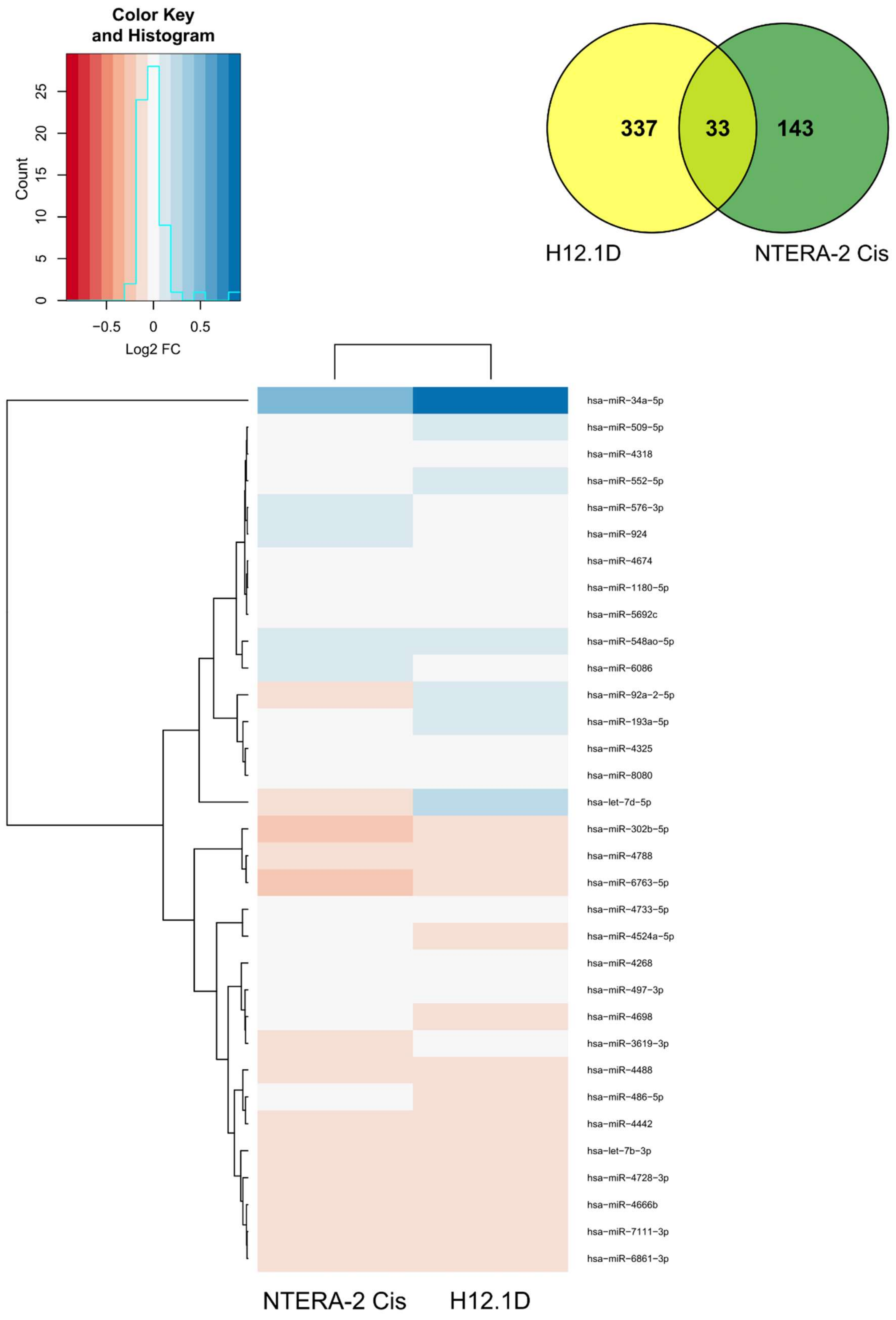

Supplementary Figure S2: Venn diagram and heatmap of overlapping miRNA in CDDP-resistant vs. -sensitive TGCT cell lines. Venn diagram analysis of two CDDP-resistant isogenic cell lines reveals 33 miRNAs whose expression was significantly changed ($p < 0.05$) in H12.1D vs. H12.1 and NTERA-2 Cis vs. NTERA-2 cell lines. The common miRNAs are presented by overlapping overexpressed miRNAs in analysed 3

TGCT cell lines: H12.1 (yellow) and NTERA-2 Cis (green). Heatmap illustrates 33 common differentially expressed miRNA with log2 transformed mean FC ($p < 0.05$) in CDDP-resistant TGCT cell lines H12.1D and NTERA-2 Cis in comparison with -sensitive parental TGCT cell lines H12.1 and NTERA-2. Red colour indicates decreased relative expression and blue colour indicates increased relative expression.

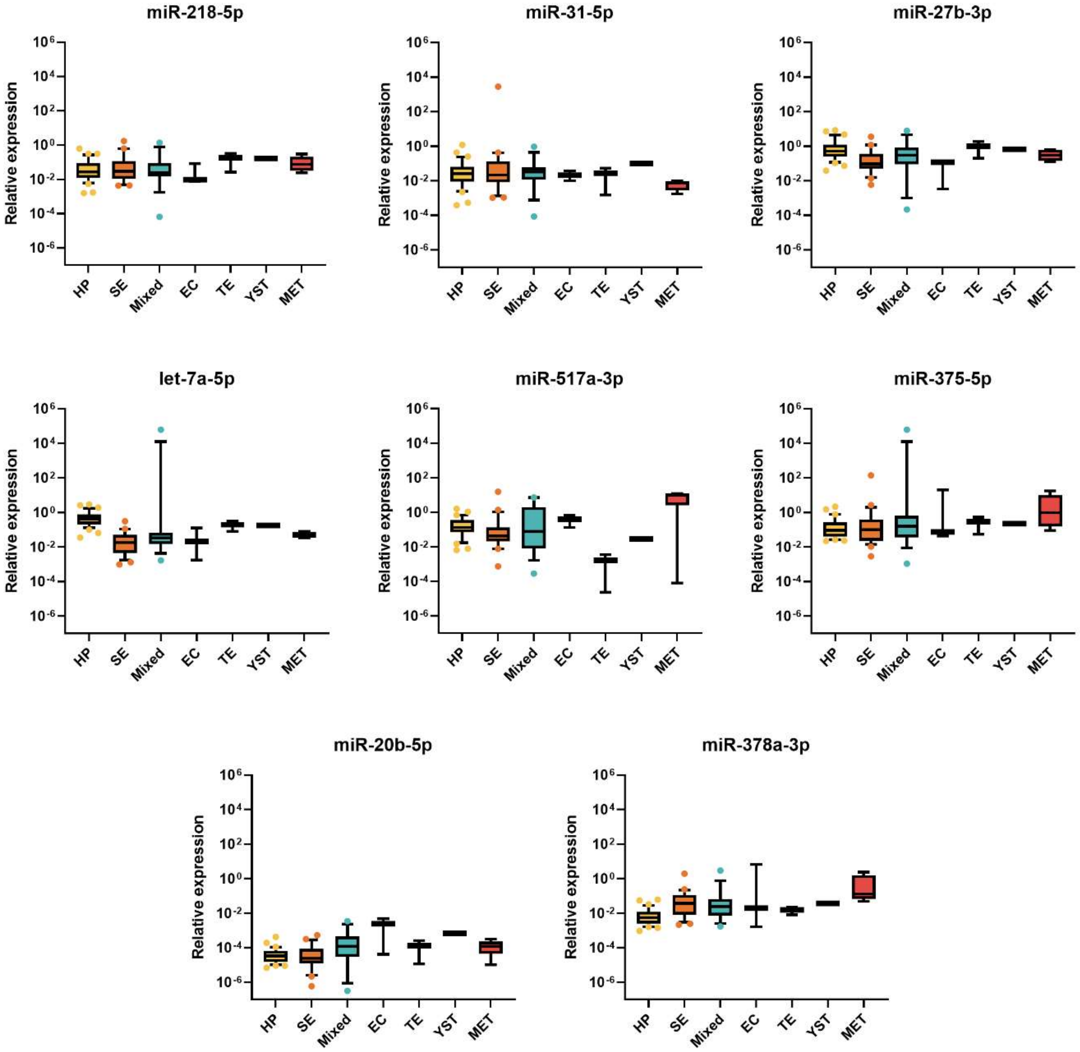

Supplementary Figure S3: Expression of miRNA in a set of patient tumour samples according to histological classification. Boxplots show relative miRNA expression (2-ΔCt) of miR-218-5p, miR-27b-3p, miR-517a-3p, miR-20b-5p, miR-31-5p, let-7a-5p, miR-375-5p and miR-378a-3p, normalized on geometric mean of SNORD44 and SNORD38B in primary tumour [seminoma (n = 23), mixed (n=18) EC (n = 4), TE (n = 2) and YST (n = 1)], metastatic (n = 5) and healthy parenchyma (n = 33) samples. Data are presented as median with interquartile range. Whiskers show 10-90 percentile. Abbreviations: Healthy parenchyma (HP), seminma (SE), embryonal carcinoma (EC), teratoma (TE), yolk sac tumour (YST), metastasis (MET).

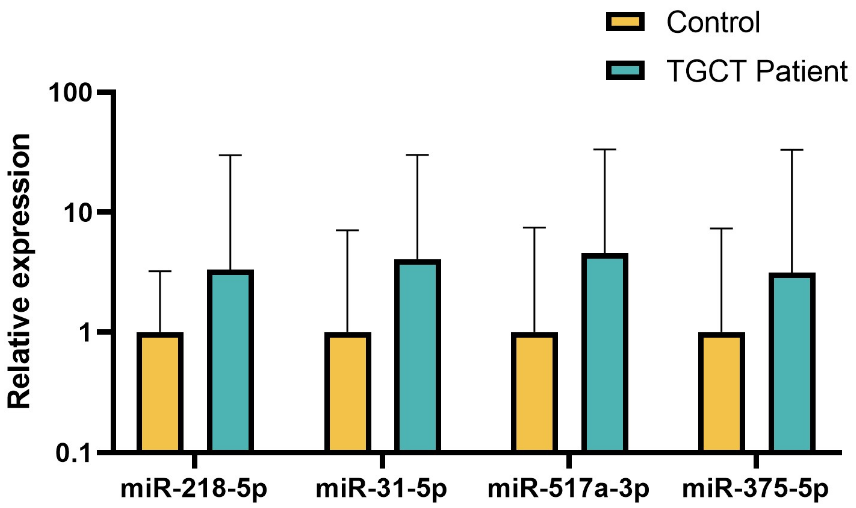

Supplementary Figure S4: Quantification of miRNA in plasma samples of TGCT patients and healthy donors. Bars represent relative miRNA expression (2-ΔCt) of miR-218-5p, miR-31-5p, miR-517a-3p and miR-375-5p, normalized on geometric mean of miR-191, SNORD44 and SNORD38B in plasma samples of TGCT patients (n = 12) and healthy donors (n = 5). Data are presented as mean FC (2-ΔΔCt) from three technical and three biological replicates. Error bars represent upper and lower limits of expression (2-ΔΔCt ± SDΔCt).

Supplementary Table S1: Overlap of the predicted target genes used for functional annotation analysis. The table lists specific pairwise overlap of predicted target genes of miR-218-5p, miR-31-5p and miR-199a-5p according to miRDB and Target Scan Human 8.0.

miR-218-5p + miR-31-5p + miR-199a-5p	miR-218-5p + miR-31-5p	miR-218-5p + miR-199a-5p	miR-31-5p + miR-199a-5p
ZBTB20	*SLC6A6*	*TOX3*	*SMARCD1*
FZD4	*SOX11*	*SHOC2*	*RAB9B*
CACUL1	*RHOBTB1*	*TSPAN5*	*PDE4D*
CEP85L	*SLC5A3*	*RFX3*	*ZDHHC21*
	RIMS3	*SLC24A4*	*PPP1R9A*
	SNTB2	*RIMS1*	*PREPL*
	SATB2	*TSPAN3*	*TENM4*
	TACC1	*RLIM*	*CRYBG3*
	SEPHS1	*WDR44*	*GPRC5A*
	SERTAD2	*RNF38*	*CACNB2*
	SPRED1	*PAN3*	*CLOCK*
	PAX5	*RASSF2*	*CBL*
	NUMB	*PPARGC1A*	*GRB10*
	NUFIP2	*KIT*	*CCNJ*
	KDM5A	*ONECUT2*	*LMAN2*
	OXSR1	*KLHL29*	
	LRRC55	*PDE7A*	
	PCDH8	*KPNA4*	
	POU2F1	*PURA*	
	PIK3C2A	*MIER3*	
	FIGN	*RBM47*	
	EGLN3	*NPAS2*	
	DPY19L3	*NAA15*	
	C11orf87	*GRIP1*	
	EIF5A2	*FLRT3*	
	DCBLD2	*HAPLN1*	
		AGO1	
		ARF6	
		CNOT6L	
		BEND3	
		EPHA7	
		FUT9	
		GPR63	
		CLCN3	
		CNN1	
		GSK3B	

Supplementary Table S2: STR profiles of TGCT cell lines used.

	**Cell line**														
**Polymorfism**	**1411HP**			**1777NRpmet**			**2102EP**			**H12.1**			**H12.1ODM**		
Amelogenin	X	Y		X	Y		X	Y		X	X		X	X	
CSF1PO	10	10		10	10		11	12		10	12		10	12	
D13S317	14	14		8	11		8	8		11	11		11	11	
D16S539	9	11		10	13		11	12		13	13		13	13	
D18S51	17	17		14	14		15	15		13	15	16	13	16	
D19S433	14	14		14	14		13	14		13	14		13	13	
D21S11	31.2	32		30	31		28	29		30	31.2		30	31.2	
D2S1338	24	25		24	25		17	20		16	20		16	20	
D3S1358	14	14		16	18		16	16		16	17	18	17	18	
D5S818	12	12		13	13		11	11		11	12		11	12	
D7S820	9	9		10	10		7	13		8	11		8	11	
D8S1179	12	13		13	16		14	15		13	14		13	13	
FGA	22	22		27	27		22	22		20	21.2	22	20	21.2	
Penta D 0 0	0	0		0	0		0	0		0	0		0	0	
Penta E	0	0		0	0		0	0		0	0		0	0	
TH01	7	9,3		6	6		9	9,3		6	9.3		6	9.3	
TPOX	8	10		9	11		8	11		8	10	11	10	12	
vWA	14	18	19	18	20		16	17	18	14	15		14	15	

Used database: Expasy – Cellosaurus

Supplemetary Table S3. Characteristics of TGCT patients and tumour samples used in this study.

Characteristic	TGCT patient
n	48
Primary tumour sample	n = 48
Metastasis sample	n = 5
Age, Years, Median	29.5
Tumour size mm (Mean ± SD)	5.03 ± 3.36
Histologic type	
Seminoma	23
Mixed	18
Embryonal carcinoma	4
Teratoma, postpubertal-type	2
Yolk sac tumour, prepubertal-type	1
Metastasis at presentation	58.33 % (n = 28)
Vascular invasion	52.08 % (n = 25)
Adjuvant chemotherapy	72.92 % (n = 35)
Vital status	
Alive, no evidence of disease	91.67 % (n = 44)
Died from disease	6.25 % (n = 3)
Alive, active disease	2.08 % (n = 1)
Disease relapse	
No	77.08 % (n = 37)
Yes	12.5 % (n = 6)
Progression	10.42 % (n = 5)
TNM classification	
I (39.58 %, n = 19)	
IA	42.11 % (n = 8)
IB	31.58 % (n = 6)
IS	21.05 % (n = 4)
II (37.5 %, n = 18)	
IIA	50 % (n = 9)
IIB	27.78 % (n = 5)
IIC	16.67 % (n = 3)
III (20.83 %, n = 10)	
IIIA	10 % (n = 1)
IIIB	60 % (n = 6)
IIIC	30 % (n = 3)
NA	2.08 % (n = 1)
IGCCCG	
Good	45.83 % (n = 22)
Intermediate	6.25 % (n = 3)
Poor	6.25 % (n = 3)
NA	41.67 % (n = 20)

n of healthy parenchyma tissue = 33, median age = 55 (14 – 87), *metastasis samples were acquired from the same patients with primary tumour – four different metastasis samples were from the same patient and one sample from another patient.

Supplementary Table S4. Characteristics of TGCT patients whose plasma samples were used in this study.

Characteristics	TGCT patient
n	12
Age, Years, Median	37
Tumour size mm (Mean ± SD)	6.04 ± 2.78
Histologic type	
Seminoma	n = 6
Mixed	n = 4
Yolk sac tumour, postpubertal-type	n = 1
Embryonal carcinoma	n = 1
Metastasis at presentation	41.67 % (n =5)
Adjuvant chemotherapy	100 % (n = 12)
Vital status	
Alive with no disease	100 % (n = 12)
Disease relapse	
No	91.67 % (n = 11)
Yes	-
Progression	8.33 % (n = 1)
TNM classification	
I (58.33 %, n =7)	
IB	85.71 % (n = 6)
IS	14.29 % (n = 1)
II (16.67 %, n = 2)	
IIA	100 % (n = 2)
III (25 %, n = 3)	
IIIA	33.33% (n = 1)
IIIB	33.33% (n = 1)
IIIC	33.33% (n = 1)
IGCCCG	
Good	25 % (n = 3)
Intermediate	8.33 % (n = 1)
Poor	8.33 % (n = 1)
NA	58.33 % (n = 7)

n of healthy donor plasma samples = 5, median age = 33 (28 – 35).
